# 3D Measurement of Neutron-Induced Tracks Using Confocal Microscopy

**DOI:** 10.3390/s25175256

**Published:** 2025-08-23

**Authors:** Gavin K. Gillmore, David Wertheim, Alan Flowers, Maria Dugdale, Jonathan S. Eakins, Kerry Olssen

**Affiliations:** 1 School of Science, Bath Spa University, Bath BA2 9BN, UK; 2 Faculty of Engineering, Computing and the Environment, Kingston University, Kingston KT1 2EE, UK; d.wertheim@kingston.ac.uk (D.W.); a.flowers@kingston.ac.uk (A.F.); 3 Radonova Scientific Ltd., Market Industrial Estate, Yatton BS49 4RF, UK; maria.dudgale@radonova.co.uk; 4 Radiation, Chemicals, Climate and Environmental Hazards Directorate, UK Health Security Agency (UKHSA), Chilton OX11 0RQ, UK; jonathan.eakins@ukhsa.gov.uk (J.S.E.); kerry.olssen@ukhsa.gov.uk (K.O.)

**Keywords:** Solid-State Nuclear Track Etch Detectors, neutrons, laser confocal microscopy

## Abstract

Using a 3D microscope imaging technique that we pioneered for alpha-track imaging of Solid-State Nuclear Track Detectors (SSNTDs), here, we present results from imaging of neutron-induced recoil proton tracks formed by exposing CR39-based detectors to an ^241^Am(Be) neutron source. Detectors were arranged at zero, thirty, and sixty degrees to the source to assess any variation in the tracks according to source orientation. An Olympus (Olympus Corporation Japan) LEXT laser scanning confocal microscope was used to image the SSNTDs. Depth and cross-sectional size measurements were made on nine tracks, with a median (range) of 3.07 μm in depth (min 0.98 μm to max 8.34 μm), width in plan view of 7.49 μm (min 4.00 μm to 14.89 μm max), and breadth in plan view of 8.41 μm (min 4.17 μm to max 11.80 μm). In this study, we have shown our confocal microscopy approach can successfully image the 3D surface of neutron-induced tracks in SSNTDs; the imaging method thus enables the measurement of track cross-sectional dimensions and depth, as well as the identification of angled tracks.

## 1. Introduction

Neutrons, from thermal up to fission-source energies, can be used for a wide variety of purposes, including the examination of geological materials [1,2]; energy production and storage [3,4,5]; assessing chemicals in the environment [6]; archaeological investigations [7,8]; and in the study of polymers and plastics [9,10]. Neutrons have also been used in various health studies [11,12]. In many of these applications, it is important to know the energy spectrum and fluence of neutrons incident on a target location. Oda et al. [13] demonstrated how CR39-based Solid-State Nuclear Track Detectors could be used for neutron spectrum measurements. According to Ziaie et al. [14] such detectors can be applied to detect small fluences of thermal and fast neutrons simultaneously (see also [15,16]). In summary, knowing the dimensions of tracks left by neutrons is useful to determine neutron energy sources and improve the accuracy of detection and spectrometry. This is useful in neutron field characterisation and astronaut dosimetry.

CR39 (an optically clear thermoset polyallyl diglycol carbonate (PADC) polymer; C_12_H_18_O_7_)-based Solid-State Nuclear Track Detectors (or SSNTDs) have been used extensively due in part to their affordability to examine cosmic rays, long-lived radioactive elements, radon concentrations in buildings [17,18,19], the age of geological samples, and for the measurement for neutrons (see [20,21,22,23,24]).

As a charged particle traverses through the polymer, it creates an ionisation trail along its path if its linear energy transfer (LET) is above a given minimum threshold. The trail or track can then be enhanced by chemical etching to expose radiation damage, as the damaged area is more sensitive to the etchant than the bulk material. This is performed using solutions of caustic alkalis, such as sodium hydroxide, at elevated temperatures for several hours. The subsequent tracks may appear as visible pits. Typically etched alpha tracks, because of exposure to radon, have an equivalent (that is measured using the same methodology/technique) diameter of between 20 and 60 μm with a depth of 10 to 20 μm, both dependent on etching conditions [19]. In this study, we utilised the same technique for examining recoil proton tracks in CR39 produced by ^241^Am(Be) neutron sources.

The application of 3D laser scanning confocal technology for imaging of nuclear tracks is a technique developed by Wertheim and Gillmore [17] and adapted to illustrate and provide detailed measurements of other materials such as volcanic ash particles [25] and other particulates (e.g., PM10s and PM2.5s) [26]. This technique allows us to scan a sample with a laser point by point, detecting the reflected light through a pinhole in front of the microscope’s light detector, to eliminate out of focus light. This ensures that the image is sharp and clear with minimal blur. The sample can be scanned in slices, adjusting the focal point and measuring the intensity of reflected light. From the collected data, a 3D image can be created. This technique allows us to quantify tracks and particles, providing, in the case of nuclear tracks, the depths, widths, morphologies, volumes of tracks, and precise track angles. These 3D images can then be animated and viewed from whatever orientation is desired. Typically, alpha and neutron tracks in SSNTDs are viewed by 2D optical microscopy, although other methods have also been utilised such as scanning electron microscope (SEM)/atomic force microscope (AFM) analyses [24]. Laser confocal imaging allows for direct, noninvasive serial optical sectioning with a minimum of sample preparation. This differs from AFM, which requires a probe to interact with the surface of the sample, and SEM analysis, where the sample must be mounted appropriately to be viewed. SEM analysis typically requires coating the sample in conductive material to prevent charging effects and improve image quality. The image produced is an electronic representation rather than a direct colour image, which is produced by scanning laser confocal imaging.

Traditional optical microscopy can be used to capture 2D images from SSNTDs such as CR39 detectors; 2D image analysis software can then be used to measure track parameters. The aim of this study was to investigate applying reflectance confocal microscopy to image the 3D surface of neutron tracks in CR39 detectors that had not been specially chemically enhanced for neutron detection. In other words, the CR39 detectors used were designed to detect both alphas and neutrons.

Neutrons, unlike alpha particles, which directly impact the CR39 detectors, polymer chains interact with the hydrogenous component in the material through secondary reactions, producing charged particles (proton recoils) that create damage trails. These damage trails in the plastic are the result of ionisation by secondary protons as they pass through the PADC. Additives to the plastic can be used to improve its response to neutrons. Our approach has the potential to help improve dose estimates and might give rise to a better ability to select higher-quality plastics for PADC dosemeters.

## 2. Methodology

The angled tracks experiments were carried out using the Secondary Standard neutron facilities of the UK Health Security Agency’s (UKHSA) Radiation Metrology group, using Radonova-sourced CR39 detectors. The room was designed to minimise scattered neutrons by using a lithium doped ‘soap’ (trade name Premadex) on the walls. In addition, the UKHSA uses a 3 mm lead shield to reduce the photon component to virtually zero. This lead shield sits around the source tube, providing full coverage of the whole tube during exposure.

The detectors were processed by chemical etching. These detectors can detect thermal, intermediate, and fast neutrons. Understanding the angular response of detectors is essential for calibrating neutron measurement systems [27].

The dosemeters (SSNTDs) were irradiated free in air at a distance of 35 cm from the centre of an ^241^Am(Be) neutron source and exposed to a 2 mSv dose equivalent, producing roughly 20 neutron tracks per mm^2^. They were mounted securely on the top of a bespoke aluminium ring tabletop in a chamber. The tabletop is made from a substance with low atomic number (Z—low Z materials have a low electron density), which may be more effective at Compton scattering, such as of the 60 keV gamma rays emitted from the shielded ^241^Am(Be) source, for example. However, whilst this could potentially be problematic for some designs of photon-sensitive detectors, the PADC used in the current measurements has no significant response to photons, so it is essentially ‘blind’ to any scattered gamma field. Minimising the amount of scattered radiation reaching the detector is beneficial in terms of radiation dosimetry, improving accuracy of results. The ring tabletop was fixed around the isotropic ^241^Am(Be) source, with the reference points defined as the centres of the dosemeters. The latter were exposed to uniform irradiation conditions.

**Figure 1:**
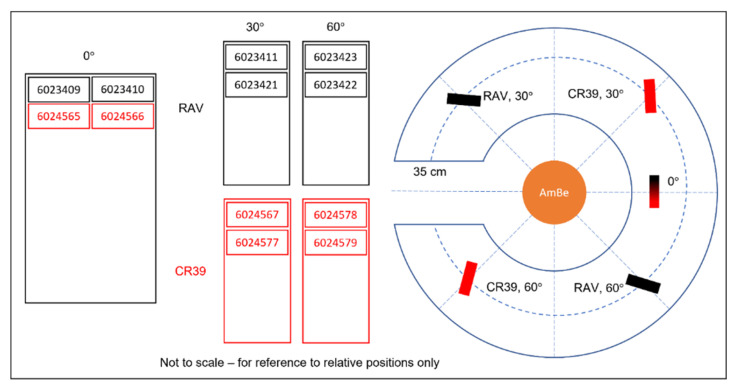

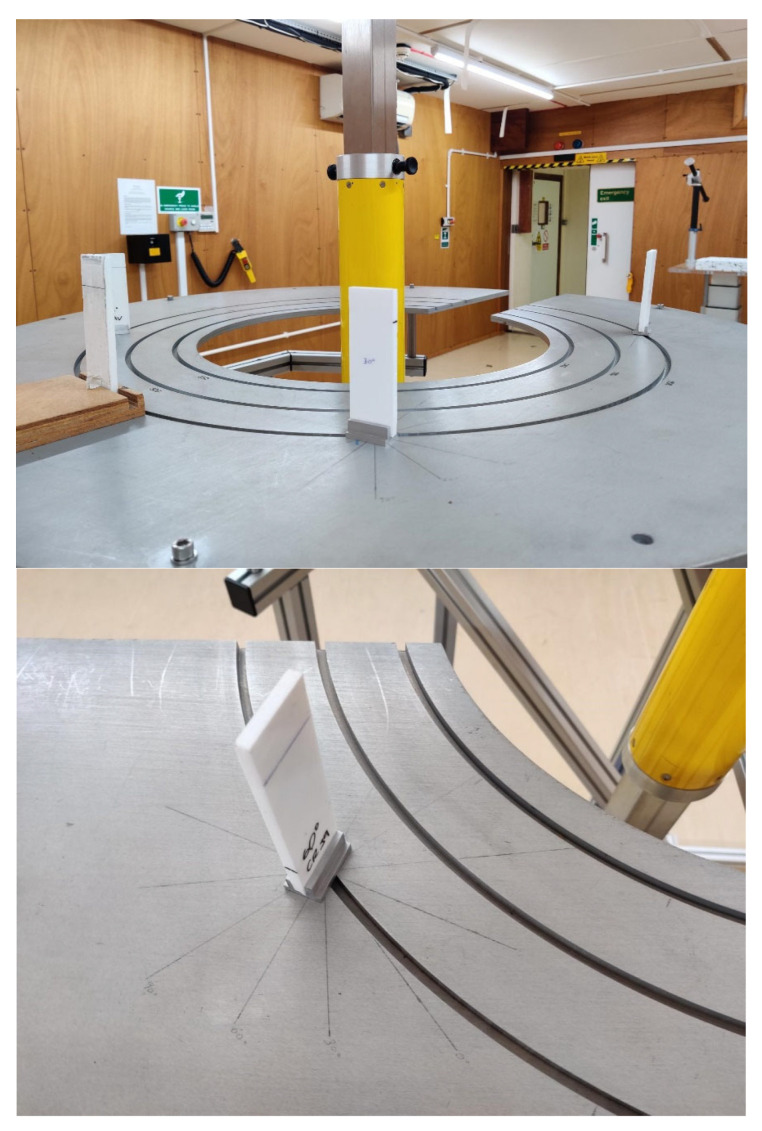
Diagrammatic and photographic explanation of the experimental set up at the UKHSA. Note that in this experiment, we compared standard CR39 detectors at 0°, 30°, and 60° angles to the source with alternative detectors (RAV) with a different plastic formulation. We do not report on the latter in this paper.

Three sets of dosemeters (four in total) were mounted at various angles: one dosemeter at 0°, one at 30°, and two at 60°. The angles were measured on the tabletop using a protractor, with a second individual performing a confirmatory check of all measurement parameters. Figure 1 shows a diagrammatic representation of the experimental set up, a photograph of the experiment, and close-up image showing how the angle between the detector and source was varied.

It should be noted that rather than being a point source, the ^241^Am(Be) neutron ‘pellet’ is an extended source with fixed dimensions spanning a few centimetres; the divergence of this physical size from a point may potentially contribute to the track structures measured by the dosemeters, although simple geometric arguments suggest that these are likely to be small.

The source pellet consists of ^241^Am that decays naturally via the emission of an alpha particle, mixed with ^9^B, which can absorb the alphas to emit neutrons with an average neutron energy of 4.2 MeV and 11 MeV maximum energy. The alpha emitter (^241^Am) is in intimate contact with the beryllium and pressed into a cylindrical form that is then double encapsulated. An Am(Be) source was chosen, as this is common usage and recommended by ISO 2001 [28] for calibration of measuring devices [29,30]. Am(Be) sources have a long half-life (433 years) and are therefore useful in providing a relatively constant neutron flux [31].

For neutron imaging the CR39 plastic SSNTDs were etched for 2 h 50 m at 85 °C in 6.25 M NaOH. Post etching, the plastics were treated with a ten-minute immersion in a 2% acetic acid stop bath, followed by rinsing in deionised water.

Etched tracks in the SSNTDs were imaged with an Olympus OLS 4100 LEXT laser confocal microscope (Olympus Corporation, Tokyo, Japan) with a 405 nm laser. For 3D surface imaging a ×50 and ×100 objective lens was used with a numerical aperture (NA) of 0.95. The SSNTDs were examined using the ‘fine’ 3D scanning mode. The top (upper slide surface) and lower track levels were determined manually.

Using this imaging technique, detailed measurements of the width, height, and depths of the etched neutron tracks could be obtained. For clarity, it should be noted that when we refer to the width (the minor axis) and breadth (the major axis) of the pit in terms of measurements here, we are referring to the plan view of the pit, as these are often not perfectly circular, not the recoil proton track length, which is represented here by depth measurements. At other angles, they will differ by the cosine of that angle. In other words, depth numbers quoted refer to the perpendicular depth from the surface of the detectors to the bottom of each etched pit. The plan view of each pit is typically elliptical; in the following, the breadth is associated with the major axis length of the ellipse, and the width its length along the minor axis.

## 3. Results

**Figure 2:**
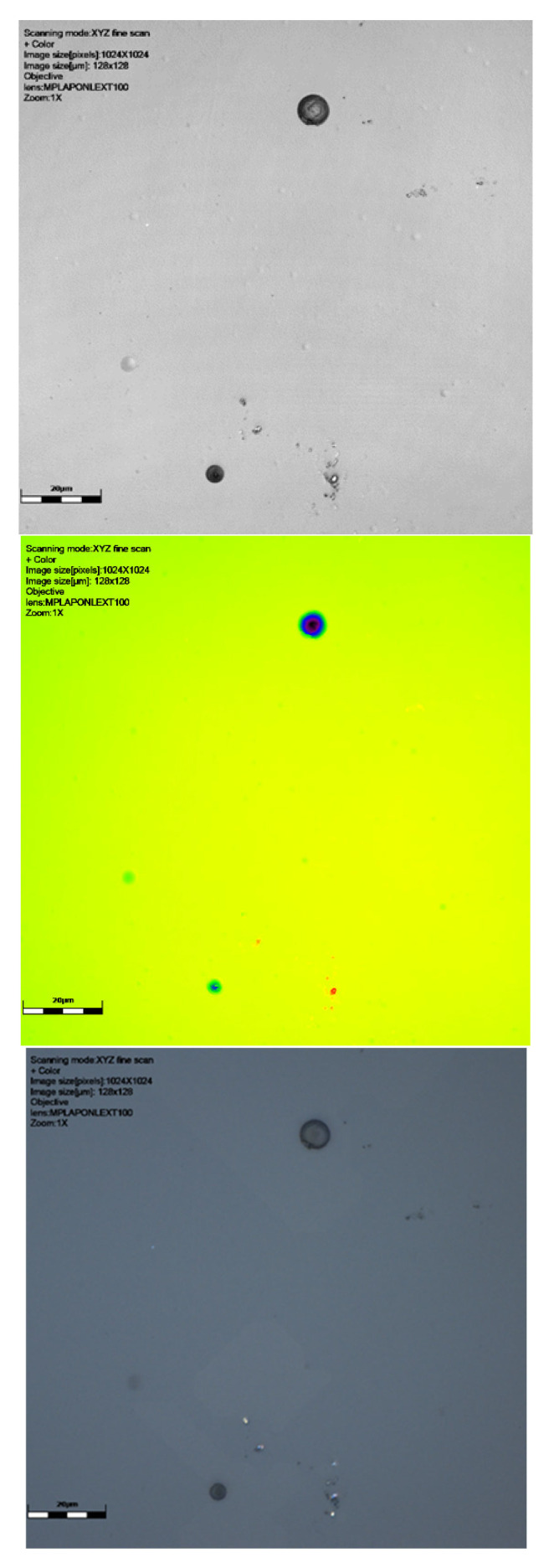

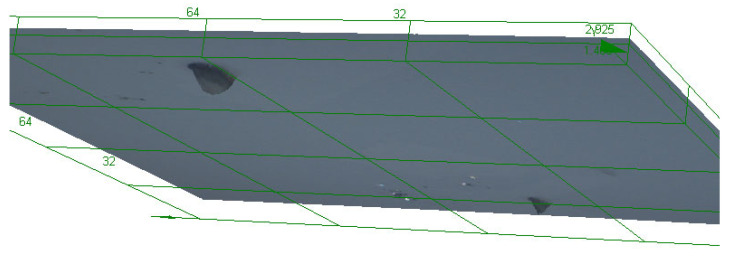
Results of scanning with LEXT confocal using ×100 objective lens: image of signal intensity, height data, and colour image (yellow green shows the surface, darkest blue the deeper track), followed by a 3D visualisation of tracks of CR39 detector 6024565 at 0° to source. The latter image is from a perspective underneath the surface of the detector. Scale bar is 20 μm.

**Figure 3:**
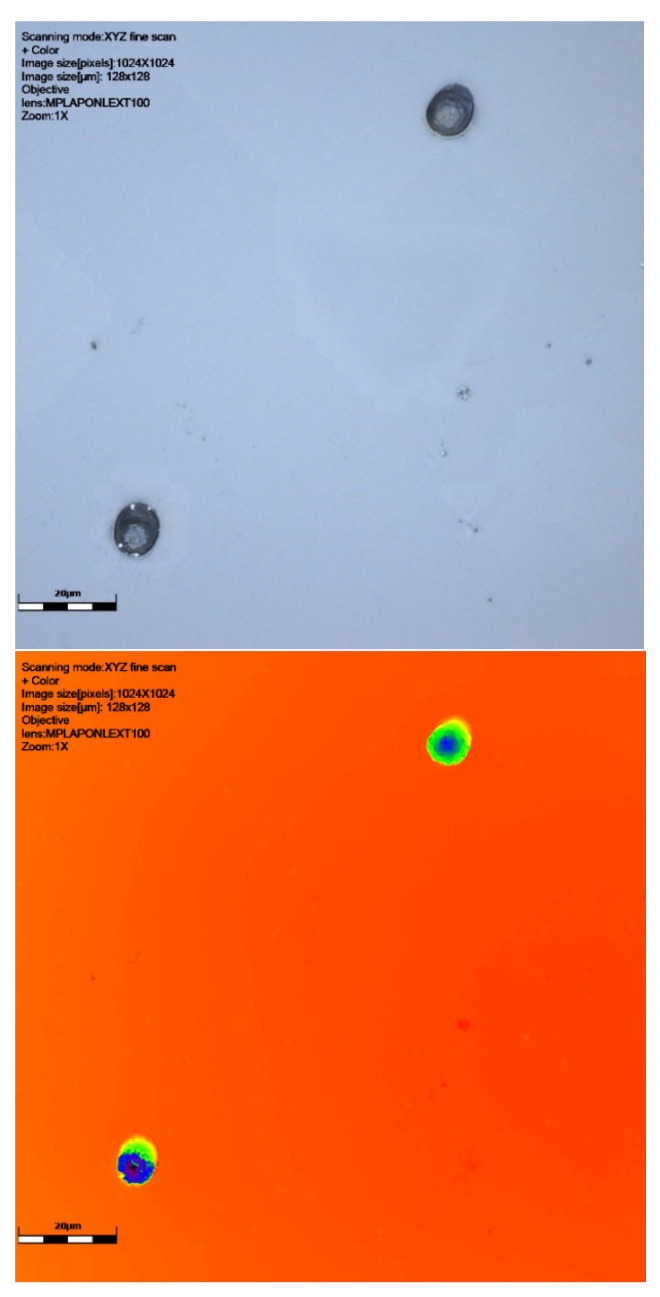

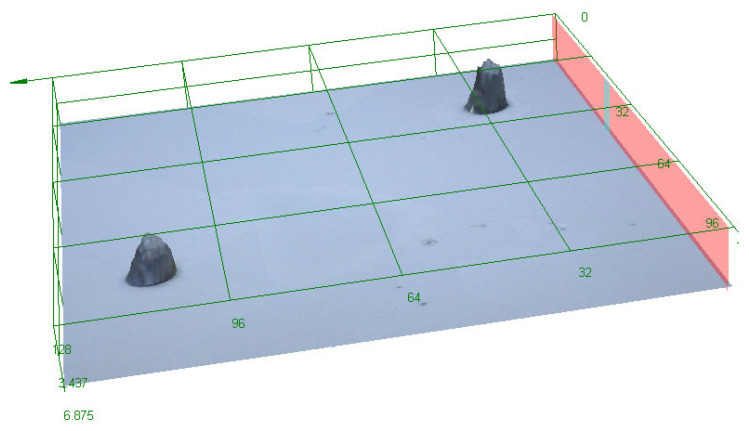
Results of scanning with LEXT confocal using ×100 objective lens: colour image and height data (orange shows surface darkest blue the deepest parts of the track), followed by a 3D visualisation of tracks of CR39 detector at 30° to source. Detector number 6024567 (Table 1). Scale bar is 20 μm.

**Figure 4:**
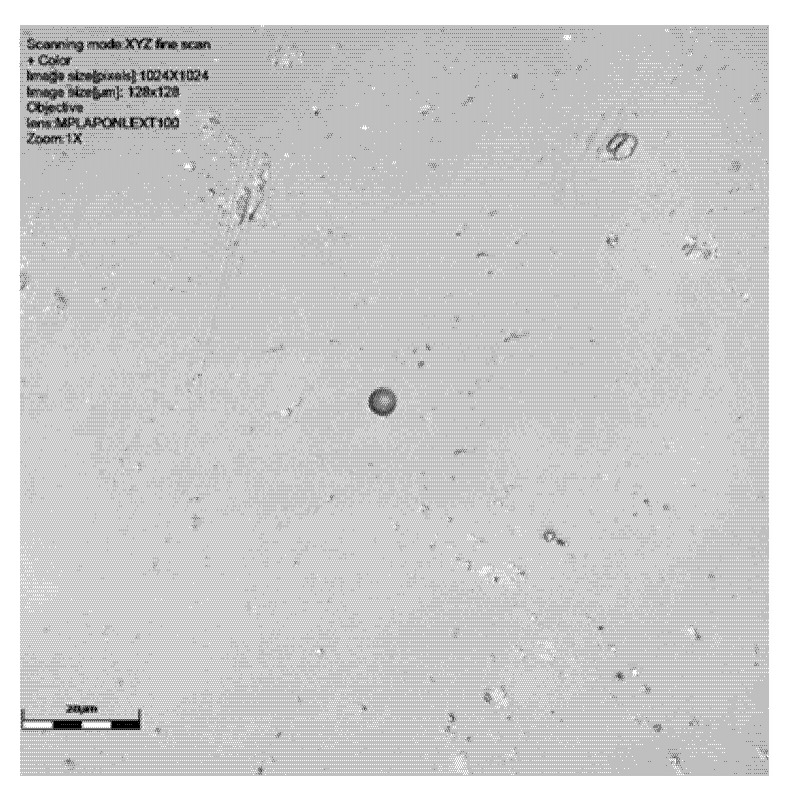

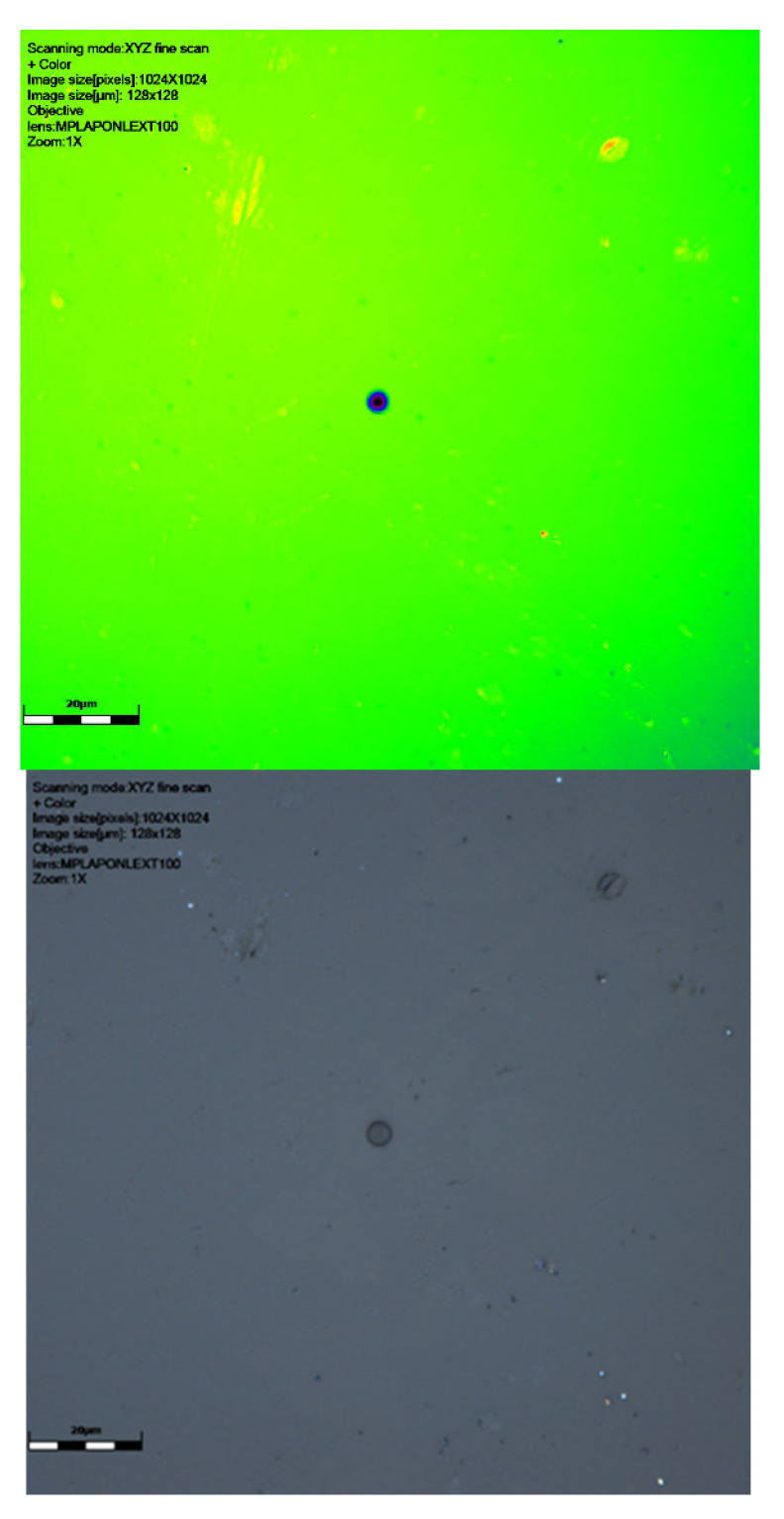
CR39 neutron track at 60° to source with ×100 objective: signal intensity, height data (green showing the surface and dark blue the track), and colour image. Detector 6024578, Table 1. This slide shows debris on the slide surface. Note the striking size difference between this track and the 6024579 detector track in Figure 5. Scale bar is 20 μm.

**Figure 5:**
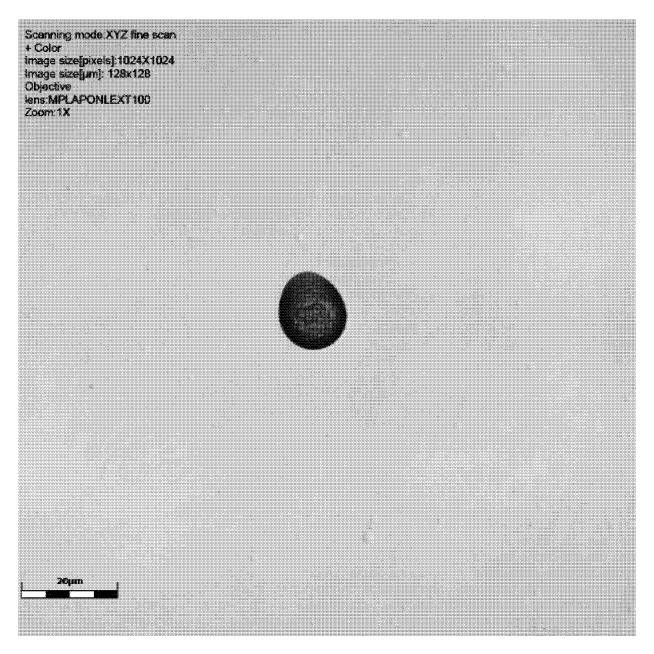

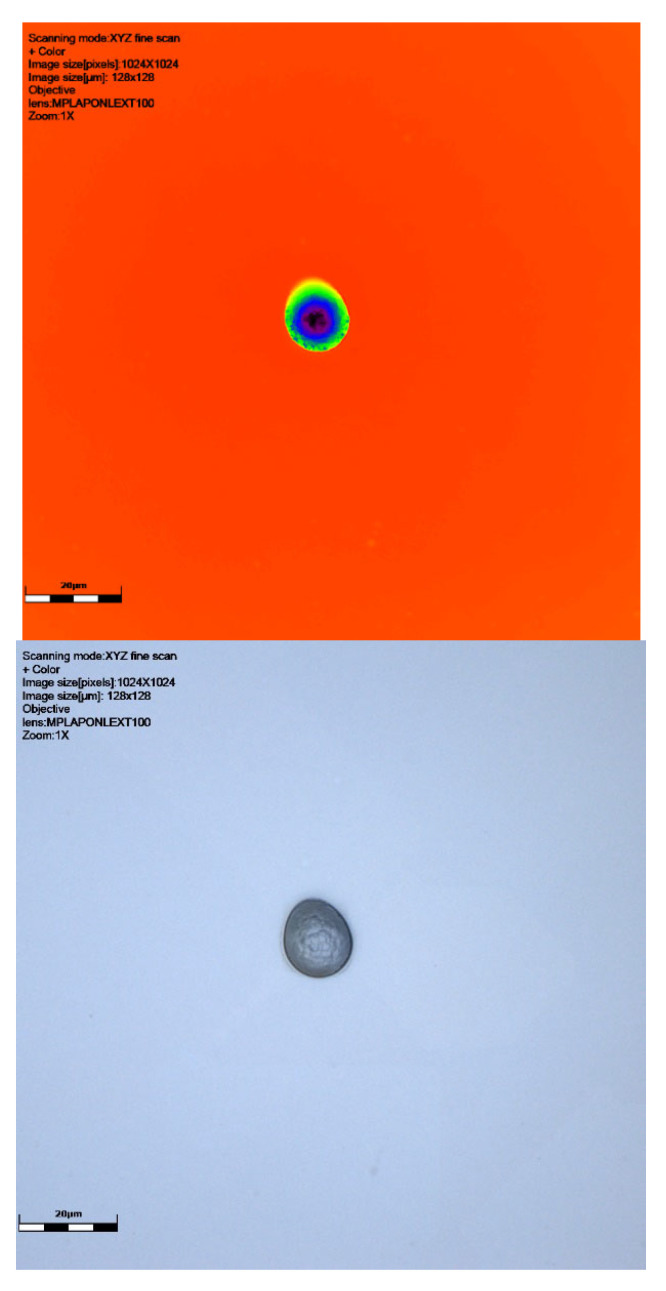
CR39 neutron track at 60° to source using ×100 objective: signal intensity, height data (orange/red shows the surface and dark blue the deepest part of the track), and colour image. Detector 6024579, Table 1. Scale bar is 20 μm.

As can be seen from Table 1 and Figure 2, Figure 3, Figure 4 and Figure 5, neutron tracks can be imaged using our technique. From Table 1, it is evidenced that two tracks were recorded on the detector at 0°, four on the detector at 30°, and two and one, respectively, on the detectors at 60°. This frequency variation is most likely a statistical effect, and no additional inferences should necessarily be drawn from it at this stage.

The results presented here are mostly smaller than the alpha tracks we have previously imaged (see [20]), as might be expected. The size ranges can be seen in Table 1. The maximum track size measured was 15.5 μm breadth and 14.89 μm width, with the smallest being 4.17 μm breadth and 4.0 μm width. Depths ranged from just 0.77 μm to 8.34 μm. The alpha tracks we have imaged previously often had depths in the order of 10–20 μm (see [20]). The median breadth for all detectors was 8.41 μm, with median width being 7.49 and depth being 3.07 μm. For detectors at different angles, the breadth range at zero degrees was 4.17 μm to 8.36 μm, width 4.00 to 7.68 μm, and depth 1.33 to 2.03 μm. At a sixty-degree angle, the breadth range was 4.67–15.5 μm, width 4.63–14.89 μm, and depth 0.99–8.34 μm (see results in Table 1).

## 4. Discussion

It is clear from Figure 2, Figure 3 and Figure 4 that our technique allows us to illustrate and measure neutron-induced recoil proton tracks in etched detectors with true 3D imaging. This has implications for fast neutron measurement in that we can measure and characterise the morphologies of even the smallest etched neutron track, down to a micron or two. This approach is not subject to issues around surface dust/particles that may impact the measurement of these smaller neutron-induced tracks or overlapping tracks that can be determined. The spectrum of recoil proton tracks extends down to the lowest energies and therefore to very short penetration tracks in the plastic. It is therefore not just dust that defines the low detection limit in CR39 neutron dosimetry, but imaging techniques are also key. It is also clear that we can illustrate the shape of neutron-induced tracks so that speciation can take place. We have demonstrated through our work on alpha tracks [18] that surface and volume imaging can be carried out on tracks in SSNTDs.

There is a potential for the increased use of neutrons in the treatment of cancer [32,33,34], which outlines the need to accurately assess and characterise neutron dose and flux. For example, Krause [32] notes that neutron brachytherapy is proving to be very useful against cancers resistant to photons and gamma rays and that the latter does not kill cancers cells as effectively as neutrons do.

Radonova Scientific suggests that for the recommended etch (the Track Analysis Systems Limited, Bristol, UK, https://www.tasl.co.uk, accessed on 13 May 2025) [35,36], tracks are typically 3–15 microns in size, overlapping with the size of alpha tracks. As can be seen in Figure 2, Figure 3 and Figure 4 and Table 1, our tracks are in line with this, being smaller than alpha tracks previously imaged by Gillmore et al. [19].

It was interesting to note that there are angled tracks on the zero-degree detectors. This is not that surprising, as there will be some small divergence of the direct neutrons and also a component from indirect neutrons scattered into the PADC by the walls of the laboratory. The same should, in theory, also be true at sixty degrees. Most recoil protons would be generated by fast neutrons, which, in theory, would arrive directionally from the source rather than a low-energy scattered component. However, the data here suggests that most of the tracks are being produced by scattered neutrons, which will be fairly isotropic, and hence, the orientation of the PADC would be irrelevant. However, the really stand out difference is in the sixty-degree results, for which the ranges of the widths, breadths, and depths was greater than at the thirty- and zero-degree angles. This result was possibly the result of one larger track, the latter bordering on what we might expect to see in an alpha size track, although the cause of this greater size is not obvious. More exposures at the different angles should therefore optimally be performed in the future to explore whether these are genuine correlations or the consequences of statistical variations within the current relatively small sample set. The precise values of the results in Table 1 and any perceived patterns in the widths, breadths, and depths exhibited within each dataset and from one dataset to another should therefore not perhaps be over-interpreted at this stage. Nevertheless, the results in Table 1 do serve to demonstrate the clear potential of the imaging techniques.

Both circular tracks and angled tracks can be imaged. As a general observation, angled tracks were smaller than the circular tracks in plan view, which is a trend also observed in alpha tracks [20].

## 5. Summary and Conclusions

In this study we investigated whether the 3D surface of etched neutron-induced recoil proton tracks in Solid-State Nuclear Track Detectors (SSNTDs) could be imaged using reflectance confocal microscopy. CR39-based SSNTDs were irradiated free in air, 35 cm from the centre of an ^241^Am(Be) neutron source and exposed to a 2 mSv dose equivalent in a bespoke aluminium ring tabletop chamber. After exposure, the detectors were processed using standard methods, and the neutron tracks were imaged using an Olympus LEXT OLS4100 microscope. Cross-sectional size as well as the depth of tracks could then be measured.

Neutron-induced tracks appear smaller than alpha tracks in SSNTDs, making them more challenging to visualise and measure. We have shown that reflectance confocal microscopy can successfully image the 3D surface of neutron-induced tracks in SSNTDs, thus enabling both the depth and track angle to be ascertained. In addition, this approach may help improved evaluation of SSNTDs with coalescing neutron-induced tracks.

## Figures and Tables

**Table 1:** Detector identification numbers, measurements for breadth, width, and depth of tracks, angle of detector to source, and medians for all tracks measured. The uncertainty of each track measured is governed by the laser wavelength and the lens numerical aperture. Lateral resolution is 0.12 microns.

Detector	Breadth	Width	Depth	Angle
	μm	μm	μm	0°
6024565	8.36	7.68	2.03	
	4.17	4.00	1.33	
				30°
6024567	11.80	9.1	4.39	
	11.09	9.03	6.14	
	6.18	5.52	0.77	
	7.22	6.35	2.26	
				60°
6024578	6.76	6.22	1.34	
	4.67	4.63	0.98	
6024579	15.5	14.89	8.34	
Median	8.41	7.49	3.07	

## Data Availability

Data are contained within the article.
